# Preparedness among dental professionals towards COVID-19 in India

**DOI:** 10.11604/pamj.2020.36.108.23694

**Published:** 2020-06-19

**Authors:** Kumar Tathagat Singh, Gaurav Mishra, Alok Kumar Shukla, Subasish Behera, Arun Kumar Tiwari, Subhasish Panigrahi, Kumar Gaurav Chhabra

**Affiliations:** 1 Department of Oral and Maxillofacial Surgery, Dr BR Ambedkar Institute of Dental Sciences and Hospital, Patna, Bihar, India,; 2 Department of Public Health Dentistry, King George’s Medical University, Lucknow, Uttar Pradesh, India,; 3 Department of Prosthodontics, Crown and Bridge and Implantology, Dental College Azamgarh, Azamgarh, Uttar Pradesh, India,; 4 Department of Conservative Dentistry and Endodontics, Hi-Tech Dental College and Hospital, Bhubaneswar, Odisha, India,; 5 Department of Prosthodontics, Crown and Bridge and Implantology, Maharana Pratap Dental College and Hospital, Kanpur, Uttar Pradesh, India,; 6 Jaiprakash Hospital and Research Centre PVT LTD, Rourkela, Odisha, India,; 7 Department of Public Health Dentistry, Sharad Pawar Dental College and Hospital, DMIMS (Deemed to be University), Sawangi, (Meghe) Wardha, Maharashtra, India

**Keywords:** COVID-19, dentistry, dental professionals, preparedness

## Abstract

**Introduction:**

novel corona virus infection has become a public health crisis leading the world to a standstill including dentistry. However, since the dental services cannot be stopped for a long period it is important that dentist be fully prepared before resuming their services. Therefore, the current study was carried out for evaluating knowledge, attitude and practices (KAP) along with perceived barriers to practice dentistry during pandemic.

**Methods:**

this cross-sectional study was conducted through an online survey questionnaire on dentists of India. Dentists were enquired for demographics, KAP and perceived barriers regarding practicing dentistry during pandemic. The knowledge was assessed based on 16 items in true or false or multiple choice questions format getting score of 1 or 0. The attitude and practices by 8 items each, on 5-point Likert scale and 4 items perceived barriers were enquired. The differences between the median scores among demographic variables were determined by applying student's t-test and keeping level of significance at below 0.05.

**Results:**

out of 500 dentists who were approached through email, a total of 296 dentists returned the questionnaire (response rate, 59.2%) among which 22 questionnaires were incomplete and thus excluded making 274 as final study participants. Overall poor median scores of knowledge and practices were obtained whereas for attitude total median score was good. Median practice scores were significantly higher among female respondents (20(6)). Median knowledge and practice scores were significantly better in study participants with age <40 years (6(4) and 19(5), respectively).

**Conclusion:**

with the recent claims of authorities that virus is going to stay in world for quite some time it is essential that dentists must be fully prepared before resuming their services and must attain proper awareness to limit the disease spread.

## Introduction

The Chinese Centre for Disease Control and Prevention on the record pronounced novel corona virus as the etiological agent of corona virus disease 2019 (COVID-19) on January 8, 2020. Beginning from Wuhan city, China, since last December the epidemics of COVID-19 have developed into a foremost challenging public health crisis globally [1,2]. The World Health Organization (WHO) affirmed COVID-19 outburst a “pandemic” on March 11, 2020 [3]. It has turned out to be a “black swan” in the language of economists, which is an infrequent and intrinsically unforeseeable incident with brutal consequences. A pandemic frequently results in worldwide recessions; moreover, the economy was already frail and in danger before the pandemic. Every facet of global trade is impacted by the virus. Nearly every sector is being affected and is bunged by this event [4]. Dental clinics have been closed ever since lockdown processes were declared in India. Most of the dentists who comprehend the corollaries of this pandemic are planning to postpone reopening of their clinics for some more time period which renders into zero earnings and subsequently a very vague prospect for dentistry and the personnel at dental clinics [5]. Owing to close proximity with infected patients, the risk of getting the infection is always higher in healthcare workers [6].

Among all healthcare professionals, dental surgeons are considered at the highest risk of contracting and transmitting the corona virus [5]. Dental services due to their unique nature like generation of aerosol, sharps handling and caregiver’s nearness to the oropharyngeal region of the patient can be attributed to these risks [1,6]. Hence, dentists have an elevated risk of taking infection from patients and probably scattering it to their near and dear ones. Furthermore, the dental clinics can most likely expose patients to cross contamination, if ample safety measures are not engaged [6]. The reactions and action plan of dental authorities globally varied from recommending dentists to stop their practices in California, USA [7]; to declining the routine examinations in the UK [8]. The dental council of India released advisory on 16^th^ April 2020 stating that dentists ought to now sternly follow all modus operandi to decontaminate, disinfect and sterilize at the dental clinics as given, permitting to treat an utmost of only 3 or 4 patients in a day. The council also urges just emergency dental treatments in the near future, additionally impacting the fiscal plight of dentists [5]. On the other hand, according to the US government COVID-19 response plan published on 13^th^ March 2020, this COVID-19 pandemic may perhaps end in over 18 months. Shutting dental practices all through the pandemic can diminish the count of affected persons; however will amplify the distress of those in need of immediate dental care [3].

Dental practitioners now face a test which they were not ready for. No management training program or dental collage could have imagined this type of situation, where there is a long-standing phase of interruption [4]. With the growing knowledge of this novel disease, dental practitioners must be better primed to recognize a likely infection of COVID-19 and should also be able to refer patients having doubtful, established, or a previous infection of COVID-19 to proper treatment centers [6]. Also, healthcare facilities are essentially necessary for any society and cannot be stopped for an extended time during such pandemic conditions [9]. As soon as the lockdown measures do alleviate up finally, dentists must practice with all precautions and protocols. Therefore, the present study was carried out for evaluating knowledge, attitude and practices along with perceived barriers by the dentist regarding various strategies for dental care provision given by competent authorities during pandemic, in a developing country like India.

## Methods

The current cross-sectional questionnaire study was carried out on the practicing dental professionals through an online survey form, in the state of Uttarakhand from 15^th^ April to 15^th^ May 2020. A close-ended structured questionnaire was prepared in English using google forms by the investigators. The dental professionals were approached with the help of social media and e-mail for filling the online survey link. Dental students and paramedical staff and were excluded. The mail ID’s of the dentists were collected from the respective Indian Dental Association (IDA) branches of the state. A reminder mail was sent after 3 days to the non-respondents, one week later a second reminder was sent and two weeks after the second reminder, the last reminder mail was sent to non-respondents. The data collection was closed on 10^th^ May 2020, after three reminders. Preceding approval to carry out the research was acquired from the institutional review board’s ethical committee. It measured the KAP of the participating dentists regarding preparedness for working during pandemic and perceived barriers for the same. The items for this instrument were generated from previous research [9] and expert opinion. A pilot study on 20 dentists was conducted prior to the final survey which analysed Cronbach’s alpha and split-half reliability values.

The modifications were done based on results and the final questionnaire had values of Cronbach’s alpha and split-half reliability as 0.81 and 0.79 for knowledge; 0.83 and 0.78 for attitude; and 0.86 and 0.80 for practice respectively. A total of 16 questions were for knowledge 8 items each for attitude and practices. The responses for knowledge were in true/false or multiple choices and each correct answer was scored as 1 and incorrect response was given score of 0. Both the attitude and practices were assessed using a 5-point Likert scale (definitely yes to definitely no and always to never, respectively). The scores for attitude ranged from 5 (definitely yes) to 1 (definitely no) and practice scores ranged from 5 (always) to 1 (never). If the obtained score was ≥80% of the possible maximum scores it was considered as good, in between range of 60 and 79% as fair and below 60% was considered as poor. Participants were also enquired for demographic details like gender, age and qualification. Differences in the median scores based upon demographics were evaluated. Furthermore, perceived barriers to practice dentistry during pandemic were also enquired.

A total of 16 questions focused on knowledge like which authority to contact in case of coming across a COVID-19 patient, mode of transmission of COVID-19, correct method to wear personal protective equipment (PPE), proper way to remove PPE, how to dispose of single use mask/PPE etc. For assessing attitude items included such as opinion on active involvement of dentists in managing COVID-19 screening, views on routinely wearing N-90 mask due to the present pandemic in dental practice and fumigating dental clinic each day to prevent the infection spread, views on need for upgrading their knowledge constantly pandemic health measures, and need for continuing dental education programs on pandemic times dental practice for dentists. Questions related to practice measured how frequently the respondents follow universal precautions of infection control on patients, use on every patient rubber dam isolation, use high-volume suction in practice, practice of asking every patient for rinsing with anti-bacterial mouthwash before treatment, practicing hand hygiene prior and after treatment and participation in training programs concerning guidelines for dental care provision during the COVID-19 pandemic.

**Statistical analysis:** the data was first entered into a Microsoft Excel version 12.0 (Microsoft, Redmond, Washington USA) and later for statistical analysis Statistical Package for the Social Sciences version 16.0 (IBM, Armonk, New York, USA) was used. To find out the relationship between the median scores and demographic characteristics student’s t-test was applied. Descriptive analysis was performed to establish perceived barriers for practicing dentistry during/after pandemic.

## Results

Out of 500 dentists who were approached through email, a total of 296 dentists returned the questionnaire (response rate, 59.2%) among which 22 questionnaires were incomplete and thus excluded making 274 as final study participants. Analysis showed that total (summation of knowledge, attitude and practices) median score of the dentists regarding preparedness of working during pandemic was moderate. Overall poor median scores of knowledge and practices were obtained whereas for attitude total median score was good (Table 1). Table 2 shows differences in median total scores and interquartile range (IQR) among various demographic variables of respondents of KAP on dental practice during pandemic. The scores were higher in dental practitioners who were less than 40 years {60(6)} as compared to those above 40 years {57(10)} and this difference was statistically significant (p<0.05). Similarly, significantly higher scores (p<0.01) were observed in practitioners who had done specialization in relationship to qualification {61(7)} than those who have not done specialization {49(9)}. Table 3 shows differences in median KAP scores (IQR) in relation to demographics of participants regarding practicing dentistry during pandemic. The difference for knowledge and attitude with respect to gender was not found significant (p>0.05) whereas the scores of practice were higher in females {20(6)} and the difference was statistically significant (p=0.037). Better knowledge and practice scores {6(4) and 19 (5), respectively} were found in participants of less than 40 years of age. Likewise, specialists showed better median knowledge and practice scores {6(4) and 20.5 (5), respectively}. Most of the respondents perceived monetary investments to continue safe dental practice as a major barrier (92.3%). The least perceived barrier was demand for dental treatment will reduce a lot due lack of insurance coverage {221 (80.6)} Table 4.

**Table 1:** the range of scores, medians and IQR obtained for the study participants in terms of total KAP

	Possible range	Obtained Range	Median	IQR
Total KAP score	16-96	31-74	56	11
Total knowledge score	0-16	0-12	6	4
Total attitude score	8-40	18-38	35	8
Total practice score	8-40	8-32	18	12

**Table 2:** demographics of respondents and relationship between the median total score

Demographic variables		Number (%)	Median total score (IQR)	p-value
Gender	Male	202 (73.7)	59 (12)	0.071
	Female	72 (26.3)	58 (13)	
Age group	<40 years	189 (69)	60 (6)	0.024*
	>40 years	85 (31)	57 (10)	
Specialist	Yes	99 (36.1)	61 (7)	0.001**
	No	175 (63.9)	49 (9)	

* p≤0.05, **p≤0.01

**Table 3:** demographics of respondents and relationship between median KAP score

Demographic variables		Median Knowledge score (IQR)	p-value	Median Attitude score (IQR)	p-value	Median practice score (IQR)	p-value
Gender	Male	7(2)	0.647	35 (3)	0.743	18 (4)	0.037*
	Female	6(4)		34 (4)		20 (6)	
Age group (in years)	<40	6 (4)	0.001**	35 (4)	0.548	19 (5)	0.001**
	>40	4 (3)		36 (3)		16 (6)	
Specialist	Yes	6(4)	0.001**	34 (3)	0.683	20.5 (5)	0.001**
	No	5 (2)		35 (4)		18 (4)	

* p≤0.05, **p≤0.01

**Table 4:** perceived barriers for practicing dentistry during pandemic

Barriers	Total	Specialist	
		Yes	No
Getting infected with COVID-19 from a patient and co-worker	239 (87.23)	95 (95.96)	144 (82.29)
Might carry the infection back to your family	224 (81.75)	92 (92.93)	132 (75.43)
Huge monetary investment to continue safe dental practice	253 (92.34)	99 (100)	154 (88.00)
Demand for dental treatment will reduce a lot due lack of insurance coverage	221 (80.66)	87 (87.88)	134 (76.57)

## Discussion

No universal course of action is in existence for dental care prerequisite for the period of any national or global disaster, epidemic and pandemic. Due to unavailability of standard protocols, dental care has entirely stopped or drastically decreased in numerous affected countries. There is uncertainty when the situation will be back to normal, the earlier global pandemic was influenza about a century ago [10] and as health services cannot be stopped for so long, one must learn to continue practice with special measures. Thus, the present study was conducted to assess KAP of dentists regarding dental services during pandemic. To the best of author’s information, this study is the first attempt to investigate knowledge, attitude and practices about dental practice during pandemic among dental professionals in India and around the world. Findings of this study revealed that participating dentists had median knowledge 6 and practice 18; suggesting information and implementation of universal precautions were disappointing. It has been previously also documented that in developing countries infection control routines have not been extensively indexed [11]. This suggests the urgent need to take compulsory courses on guidelines issued by the competent authorities. In view of the fact that the principal route for transmission of corona virus is from aerosols and droplets [12], the chances of dental healthcare workers of exposing to infection is enhanced.

Dentists must be fully prepared before resuming complete services. Findings also revealed that attitude scores were high, which is a constructive sign that respondents may be more approachable concerning training [13]. They can make significant contributions even in the patient screening programs if trained adequately. The higher scores for knowledge and practices were found in the specialist and younger dentists (Table 3). This could be due to the fact that the personality trait of “openness” typically enhances when a person is in 20s and then gradually declines after that [14]. Furthermore, in this time of social distancing, learning the preventive measures for the COVID-19 infection is by the use of digital platform. It has been stated that younger dentists are more adoptive to digital technologies (including learning) and have experience with digital learning in their dental education [15]. Majority of study participants (92.3%) perceived monetary investments to continue safe dental practice as a major barrier. In a country where oral health awareness is low and economy is poor, the cost of dental treatment cannot be increased.

In such scenario the burden of bearing the cost of PPE for safe practices falls on the dentists. Special policies must be ensured by the government to avail these equipments for the dentist at subsidized rates. Around 87% dentists feared of getting infected with disease and 81% feared transmitting it to the family members (Table 4). These findings were in accord with another study which assessed fear and anxiety among dentist during COVID-19 [9]. At present, in the whole world, various regulatory bodies are advising the dentists to carry out merely emergency treatments. In order to stay calm and work capably, it is critical that mental coping strategies are practiced in this phase of panic and apprehension. By the meticulous following of appropriate suggestions given by the regulatory authorities this fear of dental professionals can be curtailed to a great extent.

**Limitations:** although the present survey used questionnaire for collecting data, which is a verified method for collecting information pertaining to inclinations, outlook and practices of participants; nevertheless, it requires vigilant data collection and interpretation [16]. Participants are actually applying knowledge to their practice cannot be foreseen, illustrating the inbuilt restrictions of researches of these kind [13]. There is a possibility that these mind-sets and practices of dentists may amend with the up-and-coming studies and possible treatment of COVID-19. Data collected only from one state of the country limits the generalizability. Hence results of the current study should be inferred cautiously and not be globalized. Definite intervention planning cannot be done by merely cross-sectional studies; however a good stage for future answers is provided by such studies [17].

## Conclusion

The consequences of COVID-19 all over the world are intensifying with each passing day and numerous dentists have either tailored their practices based on suggested strategies to emergency treatment only, or have shut down for an indecisive time. There is no second opinion that in the current circumstances, priority is set to dental treatments categorized as emergencies by the WHO. However, now with the claims of authorities that virus is going to stay in world for quite some time it is essential that dentists must be fully prepared before resuming their services and must attain proper awareness to limit the disease spread.

### What is known about this topic

- As the knowledge of this new disease is growing, dental practices should be better primed to recognize a likely COVID-19 infection.

### What this study adds

- As soon as the lockdown measures do alleviate up finally, dentists must practice with all precautions and protocols;
- The present study was conducted to assess the awareness, attitude, perceived barriers and practices by the dentist regarding various strategies for dental care provision given by competent authorities during pandemic, in a developing country like India;
- Can be useful for the dental practitioners in Africa and also in the whole world.
